# Correction to “Synthesis of Highly Porous 3D Cerium Oxide Networks Designed for Catalytic Applications”

**DOI:** 10.1021/acs.cgd.4c01361

**Published:** 2024-11-01

**Authors:** Jonas Lumma, Tim Tjardts, Erik Greve, Lena M. Saure, Salih Veziroglu, Rainer Adelung, Lorenz Kienle, Niklas Wolff, Fabian Schütt

**Affiliations:** † Functional Nanomaterials, Department of Materials Science, Kiel University, 24143 Kiel, Germany; ‡ Chair for Multicomponent Materials, Department of Materials Science, Kiel University, 24143 Kiel, Germany; § Kiel Nano, Surface and Interface Science KiNSIS, Kiel University, 24118 Kiel, Germany; ∥ Synthesis and Real Structure, Department of Materials Science, Kiel University, 24143 Kiel, Germany

The second author’s name, Tim Tjardts, was misspelled on the original version of this manuscript and Supporting Information. This mistake has been corrected here.

## Supporting Information Available

Evaluation of specific surface area, SEM images for different reaction times and concentrations, film thickness and weight for different concentrations; XPS widescreen, elemental composition calculated from XPS; list of used chemicals; list of used devices (PDF)

