# Supplementary material for: Correction to “Synthesis of Highly Porous 3D Cerium Oxide Networks Designed for Catalytic Applications”
Source: Cryst Growth Des. 2024 Nov 1;24(22):9848. doi: 10.1021/acs.cgd.4c01361 (PMC11583198; doi:10.1021/acs.cgd.4c01361)
Supplement: Supplementary file 1 — cg4c01361_si_001.pdf [file cg4c01361_si_001.pdf]

Supporting Information:

Synthesis of highly porous 3D cerium oxide networks designed for catalytic applications

Jonas Lumma[1*], Tim Tjardts[2], Erik Greve[1], Lena M. Saure[1], Salih Veziroglu[2,3], Rainer Adelung[1,3], Lorenz Kienle[3,4], Niklas Wolff[3,4*], Fabian Schütt[1,3*]

[1]*Functional Nanomaterials, Department of Materials Science, Kiel University, 24143 Kiel, Germany*

[2]*Chair for Multicomponent Materials, Department of Materials Science, Kiel University, 24143 Kiel, Germany*

[3]*Kiel Nano, Surface and Interface Science KiNSIS, Kiel University, 24118 Kiel, Germany*

[4]*Synthesis and Real Structure, Department of Materials Science, Kiel University, 24143 Kiel, Germany*

[*]*corresponding authors: fas@tf.uni-kiel.de, niwo@tf.uni-kiel.de, jlu@tf.uni-kiel.de*

Supplementary Note 1. Evaluation of the specific surface area of Aero-$Ce_{1-x}Zn_xO_{2-\delta}$ and t-ZnO networks.

With standard techniques e.g BET the specific surface area of lightweight framework structure with a high free volume (>90%) is hard to measure, because the amount of material per volume and thus the amount of surface per volume is low. However, the surface area of Aero-$Ce_{1-x}Zn_xO_{2-\delta}$ can be estimated with the following calculation. This type of calculation has been used before e.g., for the determination of the surface area of graphene foams grown by nickel foam assisted CVD.[1]

1. The density of a ZnO template is □0.3 g cm^{-3} with a porosity of □94 %
2. For 1 cm^3 the mass of ZnO is 0.3 g.
3. The filled volume can be determined by dividing through the density of ZnO (□5.61 g cm^{-3}) and is □ 0.053 cm^3.
4. The t-ZnO-template consists of massive ZnO rods with a mean diameter of □ 3 μm and length of 27 μm. Each ZnO rod has a volume of □ $1.908 * 10^{-10}$ cm^3.
5. The total number of ZnO rods can be determined by dividing the volume of ZnO in the template by the volume of a ZnO rod and is □ $2.8 * 10^{-10}$.
6. Assuming a smooth surface, each rot has a surface area of □ $2.615*10^{-10}$ m^2. This results in a ZnO template surface area of □ 0.073 m^2 cm^{-3} and a specific surface area of 0.244 m^2 g^{-1}.
7. Because Aero-$Ce_{1-x}Zn_xO_{2-\delta}$ is based on the ZnO templates, consisting of hollow tubes instead of ZnO rods, the number of tubes should be equal to the number of rods. Since the tubes are hollow, the surface area for Aero-$Ce_{1-x}Zn_xO_{2-\delta}$ can be doubled.
8. The volumetric surface area for Aero-$Ce_{1-x}Zn_xO_{2-\delta}$ is therefore 0.1465 m^2 cm^{-3}. The surface area can be normalized by the density of the Aero-$Ce_{1-x}Zn_xO_{2-\delta}$, which varies from □ 12 mg cm^{-3} up to □ 465 mg cm^{-3} and is between 0.3 m^2 g^{-1} and 12 m^2 g^{-1}. However, the SEM images of Aero-$Ce_{1-x}Zn_xO_{2-\delta}$ show a rather rough surface structure, which increases the surface in comparison to a flat tube surface. Therefore, the calculated surface areas should represent a lower limit.

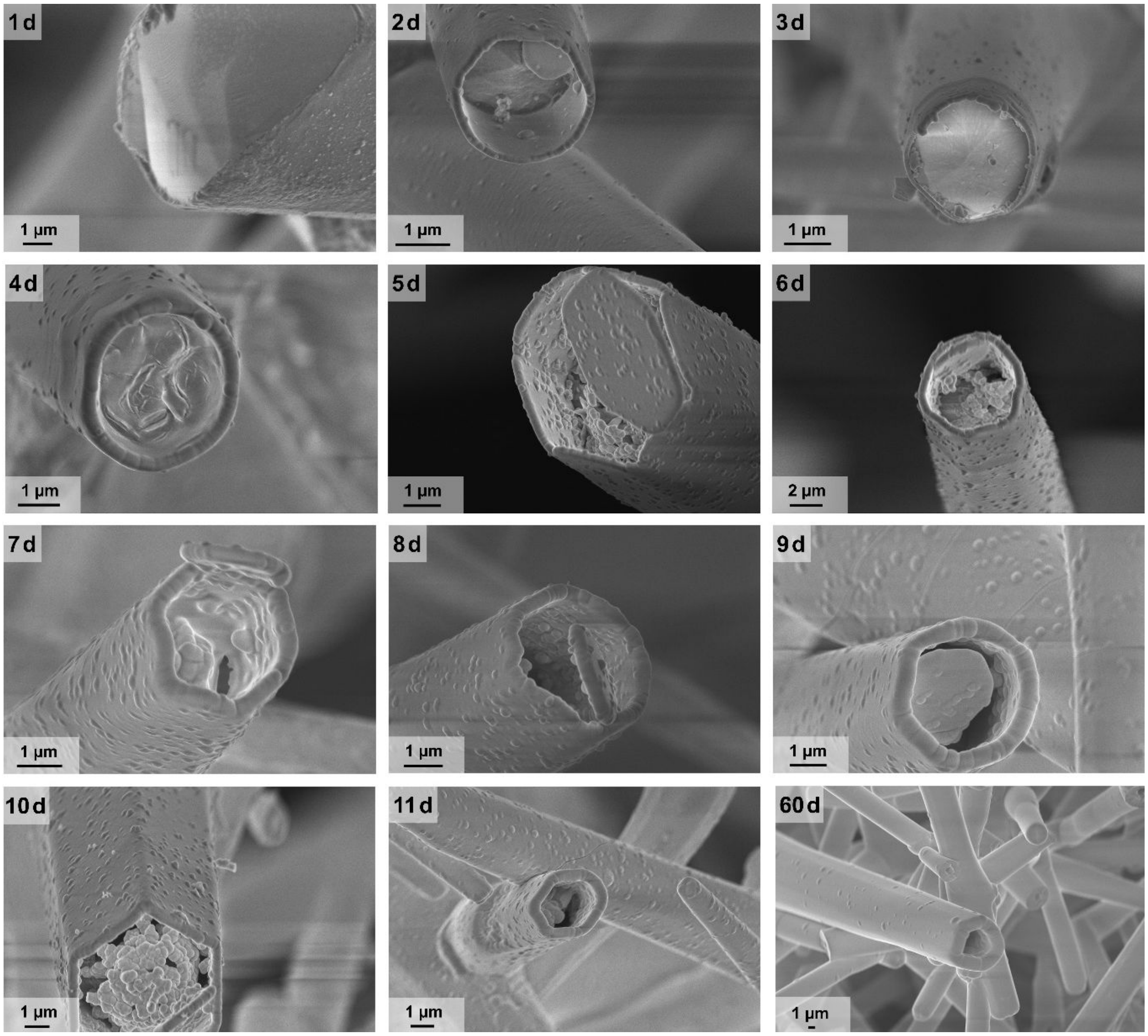

Figure SI 1: SEM images of t-ZnO covered with a film of $Ce_{1-x}Zn_xO_{2-\delta}$ after reaction times from 1 day to 11 days and after 60 days in 0.05 M cerium nitrate precursor solution.

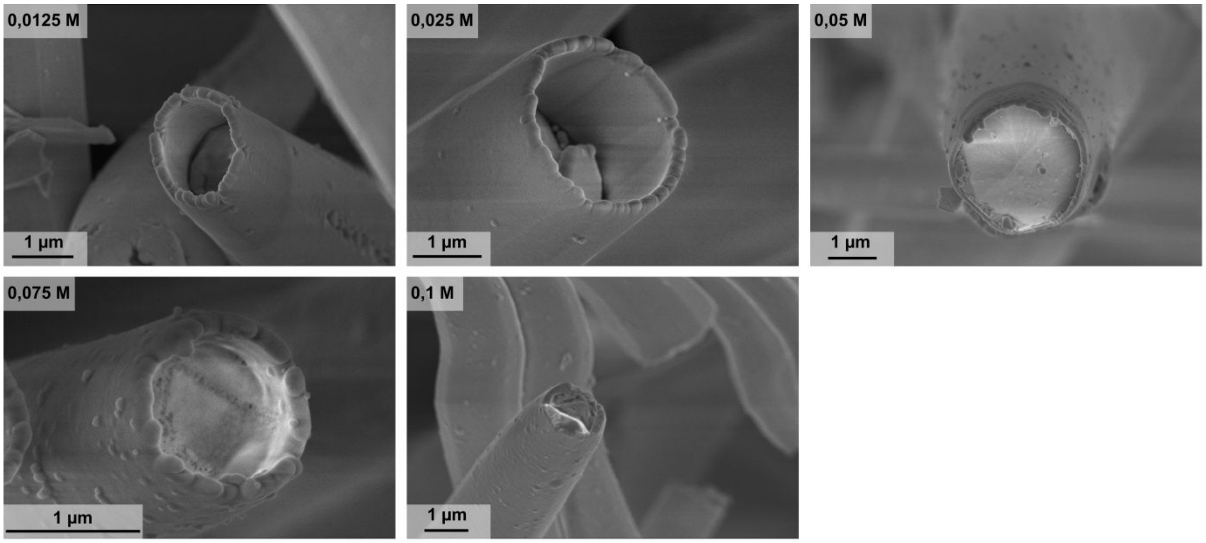

Figure SI 2: SEM images of t-ZnO covered with a film of $Ce_{1-x}Zn_xO_{2-\delta}$ after reaction times of 3 days with different precursor solution concentrations.

Film Thickness and Weight for different concentrations

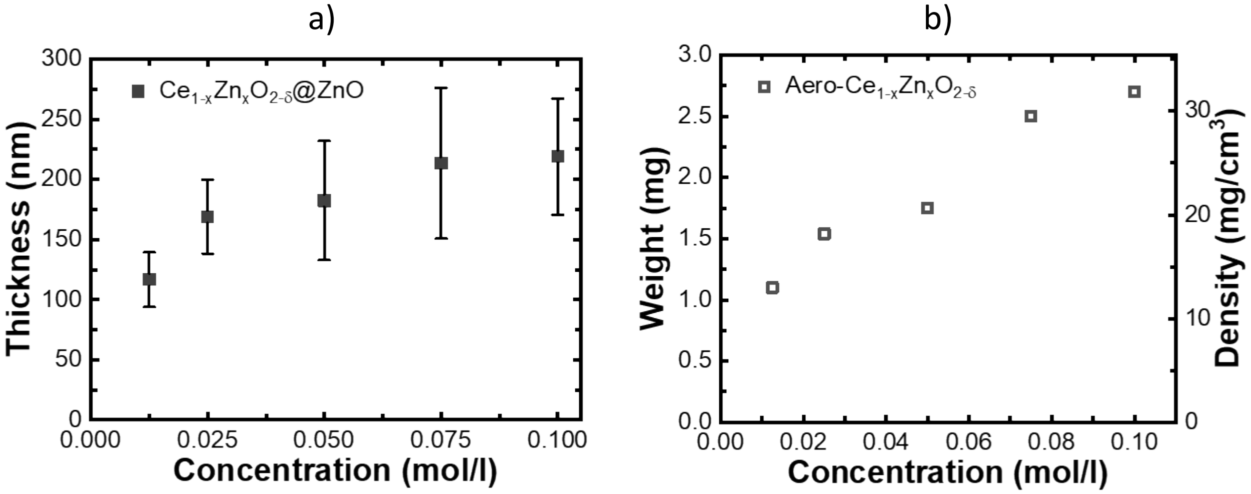

Figure SI 3: Adjustability of Aero-$Ce_{1-x}Zn_xO_{2-\delta}$ wall thickness through different concentrations. a) Thickness of Aero-$Ce_{1-x}Zn_xO_{2-\delta}$ depending on the reaction time b) and weight and density of cylindrical (height 3 mm; radius 3 mm) Aero-$Ce_{1-x}Zn_xO_{2-\delta}$ samples.

XPS Widescreen

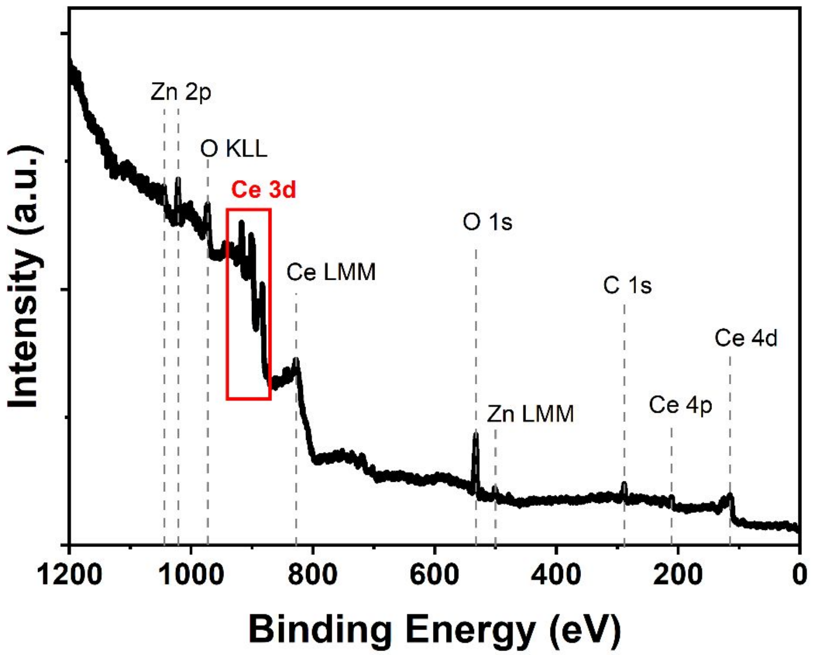

Figure SI 4: XPS Wide scan of an Aero-$Ce_{1-x}Zn_xO_{2-\delta}$ sample with indicated elemental peaks and Auger lines; the presence of Ce 3d is marked in red.

Table SI 1: Elemental Composition of the Aero-$Ce_{1-x}Zn_xO_{2-\delta}$ calculated from integration of XPS peaks under the consideration of the respective sensitivity factors.

Element	Peak used for Analysis	Relative Amount/at%
C	C 1s	18.8
O	O 1s	51.3
Ce	Ce 3d	22.0
Zn	Zn 2p	7.9

***Table SI 2**: List of used chemicals.*

Chemical	Producer	CAS Number
Cerium(III) nitrate hexahydrate	Sigma-Aldrich	10294-41-4
Polyvinylpyrrolidone	Sigma-Aldrich	9003-39-8
Ethylene glycol	Carl Roth GmbH & Co. KG	107-21-1
Rhodium(III) chloride hydrate	Sigma-Aldrich	10049-07-7
Tetrapodal zinc oxide	CAU Kiel	-
Acidic acid 100 %	Carl Roth GmbH & Co. KG	64-19-7
Ethanol	Carl Roth GmbH & Co. KG	64-17-5
Abs. Ethanol	Carl Roth GmbH & Co. KG	64-17-5
Acetone 99,5 %	Carl Roth GmbH & Co. KG	67-64-1
Propylen oxide	Sigma Aldrich	75-56-9
Citric Acid	Sigma Aldrich	77-92-9

***Table SI 3**: List of used devices.*

Device	Producer	Name
Scanning Electron Microscope	Zeiss	Ultra 55 Plus
Transmission Electron Microscope	FEI	Tecnai F30
	JEOL	ARM200F NEOARM

XRD	Rigaku	Smartlab
Oven	Carbolite	Gero 30-3000˚C
Critical Point Dryer	Leica	CPD 300
Heating Plate /Magnet Stirrer	IKA	C-MAG HP7
pH Meter	Mettler Toledo	FiveGo
Reflux Condenser	Julabo	F25
XPS	Prevac Sp. z o. o.	XPS UHV system
Flow transmitter	Festo	SFTE-5U-Q4-B-2.5K
Manometer	PCE Instruments	PCE-PDA 1L

References

[1] Chen, Z.; Ren, W.; Gao, L.; Liu, B.; Pei, S.; Cheng, H.-M. Three-dimensional flexible and conductive interconnected graphene networks grown by chemical vapour deposition. *Nature materials* 2011, *10* (6), 424–428. DOI: 10.1038/NMAT3001. Published Online: Apr. 10, 2011.
